# Determination of Pesticide Residue Levels and Serum Paraoxonase 1 Protein Levels in Obese Children: A Case–Control Study

**DOI:** 10.3390/biom16030439

**Published:** 2026-03-14

**Authors:** Nihal Inandiklioglu, Asli Atasoy Aydin, Ismail Ethem Goren, Adem Yasar, Nebile Daglioglu

**Affiliations:** 1 Department of Medical Biology, Faculty of Medicine, Yozgat Bozok University, 66200 Yozgat, Türkiye; 2 Department of Forensic Toxicology, Institute of Forensic Sciences, Ankara University, 06620 Ankara, Türkiye; asliatsy@gmail.com (A.A.A.); ismailethemgoren@gmail.com (I.E.G.); nebiled@hotmail.com (N.D.); 3 Department of Pediatric Allergy and Immunology, Faculty of Medicine, Manisa Celal Bayar University, 45030 Manisa, Türkiye; adem.yasar@cbu.edu.tr

**Keywords:** obesity, organochlorine pesticides, organophosphorus pesticides, paraoxonase 1

## Abstract

Background: Childhood obesity is becoming an increasingly prevalent global health issue. Pesticides, which pose significant threats to human health and the environment are major risk factors for various diseases, including cancer, obesity, diabetes, autoimmune disorders, and food allergies. Paraoxonase 1 (PON1) is an enzyme found on high-density lipoproteins (HDL) in serum, which hydrolyzes toxic oxon metabolites of organophosphate pesticides, certain carbamates, aromatic and aliphatic lactones, aromatic esters, and oxidized lipids through its calcium-dependent glycoprotein structure. This study aimed to evaluate the relationship between environmental pesticide exposure, childhood obesity, and PON1 levels. Methods: The study included 58 obese children with a body mass index above the 95th percentile and 43 healthy children of the same age group. Serum PON1 levels were measured using the ELISA method. Levels of polychlorinated biphenyls (PCBs), organochlorine pesticides (OCPs), and organophosphorus pesticides (OPPs) in the blood were determined through LC/MS/MS and GC analysis methods. Results: According to ELISA analysis, the PON1 level was significantly lower in the obesity group (102.8 ± 12.49 ng/mL) compared to the control group (134.8 ± 14.29 ng/mL) (*p* < 0.001). LC/MS/MS and GC analyses showed significantly higher levels of Σ_4_OPPs and Σ_4_PCBs in obese children compared to the control group (*p* < 0.001). However, no significant difference was observed between the two groups in terms of Σ_4_OCP levels (*p* > 0.05). Conclusions: Our findings highlight the presence of OPPs and PCBs in the blood of obese children. Although these factors are associated with PON1, further research is needed to evaluate their potential role as risk indicators for obesity.

## 1. Introduction

Obesity is a chronic disease associated with various conditions, including diabetes, cardiovascular disease, respiratory disorders, and cancer, and it continues to be a growing global problem [1]. In 2016, the World Health Organization (WHO) reported that over 650 million adults and 340 million children and adolescents were overweight or obese [2]. Numerous studies have shown that factors such as genetic predisposition, lack of physical activity, excessive food intake, and socioeconomic and cultural environments contribute to the development of childhood obesity [3,4].

Recent research has explored the role of obesogenic endocrine disruptors in the development of obesity, with particular attention on the impact of pesticides [5]. Pesticides are a diverse group of chemicals that provide public health benefits, such as increased food production and control of food- and vector-borne diseases. However, depending on the type of agent and exposure, they can pose significant health risks. Various pesticide compounds are used in agriculture to eliminate competing organisms, and over 20,000 products are utilized as insecticides, herbicides, rodenticides, fungicides, nematicides, tree protectors, plant growth regulators, and fumigants. These products are present in water, food, homes, schools, gardens, and workplaces. Although pesticides are designed to target specific harmful species, they may have adverse effects on human health, particularly in infants and young children, which remain incompletely understood [6]. Exposure to pesticides has been linked to health issues such as cancer [7], reproductive disorders [8], and obesity [9]. Organochlorine pesticides (OCPs), a class of persistent organic pollutants (POPs), have been widely used in agricultural pest control and disease vector management [10]. POPs, including compounds like polychlorinated biphenyls (PCBs), dichlorodiphenyltrichloroethane (DDT) and its metabolite dichlorodiphenyldichloroethylene (DDE), and hexachlorobenzene (HCB), are synthetic chemicals characterized by slow degradation and their ability to accumulate in human adipose tissue [11,12].

While factors such as genetic predisposition, excessive calorie intake, and lack of physical activity are well-known contributors to obesity [13], they do not fully explain the obesity epidemic. In recent years, the role of obesogenic endocrine disruptors, including phenols, phthalates, and pesticides, has become a focus of research [14,15]. Among pesticides, organophosphate pesticides (OPPs) and carbamates are the most widely used globally [16]. Developed in the 1930s as insecticides to replace OCPs, OPPs now account for 34% of the global pesticide market. They are widely used in agriculture and gardening [17], and their prevalence has been linked to metabolic disorders, including obesity [18].

Paraoxonase 1 (PON1) is a calcium-dependent glycoprotein enzyme associated with high-density lipoprotein (HDL) in serum. PON1 hydrolyzes various substrates, including toxic oxon metabolites of organophosphate insecticides (e.g., parathion, diazinon, chlorpyrifos), nerve agents (e.g., sarin, soman, tabun), aromatic esters (e.g., phenyl acetate), and several aromatic and aliphatic lactones (e.g., homogentisic acid lactone, dihydrocoumarin, γ-butyrolactone, and homocysteine thiolactone) [19]. PON1 exhibits paraoxonase, arylesterase, and lactonase activities [20] and protects low-density lipoprotein (LDL) and HDL from oxidative damage by hydrolyzing phospholipid hydroperoxides. It has also been implicated in diseases associated with oxidative stress, such as obesity and cardiovascular disease [21].

Given the relatively recent evidence linking obesity and pesticide exposure, further research is essential to better understand this relationship. In this study, we aimed to evaluate the association between environmental pesticide exposure, childhood obesity, and PON1 levels.

## 2. Materials and Methods

### 2.1. Study Population

This study was performed in line with the principles of the Declaration of Helsinki. Approval was granted by the Ethics Committee of University Çukurova (2 April 2021/110-10). Written informed consent was obtained from each participant. Fifty-eight obese children with a body mass index above the 95th percentile and forty-three healthy children in the same age group were included in this study. Patients with chronic diseases such as diabetes, inflammatory diseases, infectious diseases and using oral antidiabetic drugs, insulin, antihypertensive drugs, and lipid-lowering drugs, were not included in the study. Body mass index was calculated by dividing body weight by the square of height (kg/m^2^). Homeostasis model assessment–insulin resistance (HOMA-IR) value was calculated as HOMA-IR = Fasting Glucose (mg/dL) X Fasting Insulin (uIU/mL)/405.

### 2.2. Chemicals

All pesticide standards and deuterated standards were purchased from Dr. Ehrenstorfer (Augsburg, Germany) and were certified for purity (higher than 98%). Methanol (MeOH) and ultrapure water suitable for LC grade were purchased from Merck (99.8–100%, Darmstadt, Germany). Oasis HLB cartridge (3 cc, 60 mg) solid-phase extraction (SPE) cartridges were purchased from Waters Corporation (Milford, MA, USA). Working solutions were prepared daily by diluting mix stock standards (10 ng/mL) and IS solution (10 ng/mL) in MeOH. Calibration standards were prepared by serial dilution of the mixed working solution in blank blood (a constant IS concentration (dimethoate-d6)), and individual concentrations ranging from 0.1 to 100 ng/mL were obtained. All stock and working solutions were stored at −20 °C in dark glass bottles.

### 2.3. Sample Preparation

Whole blood samples of 3 mL were collected from each participant into EDTA tubes. A 1 mL aliquot of blood was transferred to a glass tube. To the sample, 3 mL of 0.1 M pH 7.4 phosphate-buffered solution and internal standard (10 ng/mL dimethoate-d6) were added. The blood sample was then centrifuged (3000 rpm, 10 min), and the supernatant was collected. Oasis HLB (3 cc, 60 mg, Waters Corporation Milford, MA, USA) cartridges were conditioned sequentially with 2 mL of methanol and 2 mL of distilled water. After sample addition, the cartridges were washed (%5 methanol), and eluents were collected with 2 mL of methanol. Eluents were evaporated under a gentle nitrogen flow until dry and reconstituted with 150 µL of MeOH. The analysis of extracts was performed using an optimized LC/MS/MS method.

### 2.4. LC-MS/MS Analysis

A total of 144 pesticides were examined in blood samples using Shimadzu 8040 LC-MS/MS (Kyoto, Japan). These analyses were separated chromatographically at room temperature at a flow rate of 0.4 mL/min on the Shim-pack column (150 × 2.0 mm ID) purchased from Shimadzu (Tokyo, Japan). All pesticides were suitably separated using the following oven program: The mobile phase consisted of 5 mM ammonium formate in water (A) and 5 mM ammonium formate in methanol (B). The gradient was started for the positive mode with 10% B and held for 10 min at a flow rate of 0.5 mL/min, and then the flow rate increased to 1 mL/min with 90% B and kept at these conditions for 5 min. After 15 min, the flow rate returned to 0.5 mL min^−1^ with 10% B. The eluent was diverted to waste for the first 1.5 min. The total run time per sample was 18 min. All the selected analytics were analyzed in positive ionization mode (ESI+). Identification and quantification were performed using two characteristic transitions for the analyzed compounds (MRM mode). The retention time, precursor ions, and product ions of 144 pesticides applied are listed in Supplementary Table S1. Calibration was performed by spiking blank blood samples with the following concentrations: 0.05, 0.1, 0.5, 1, 2.5, 5, 10, 25, 50, 80 and 100 ng/mL. The deuterated internal standard was added to blood (10 ng/mL, dimethoate-d6). The limit of detection (LOD) and the limit of determination (LOQ) were calculated based on the signal-to-noise ratio 3 and 10, respectively. The LOD was in the range of 0.01–0.02 ng/mL of blood, and the LOQ was in the range 0.05–0.18 ng/mL of blood for LC/MS-MS analysis.

### 2.5. Gas Chromatographic (GC) Analysis

Gas Chromatographic (GC) analysis was performed using an Agilent 6890N Model Gas Chromatograph (Agilent Technologies, Cheadle, UK) equipped with an ECD detector. Chromatographic determination of OCs was carried out using a 60 m × 0.32 mm D.I. fused silica capillary column HP-5 from Hewlett-Packard (Agilent, Santa Clara, CA, USA). The operating conditions were as follows: injector temperature 2500 °C; detector 3000 °C; column 70–2800 °C (2 min at 700 °C; 250 °C/min from 70 to 1500 °C; 30 °C/min from 150 to 2000 °C; 80 °C/min from 200 to 2800 °C; 10 min at 2800 °C). The carrier gas was helium. For the investigation of pesticide retention times with gas chromatography, standard pesticide solutions were prepared with hexane. Excellent linearity was obtained in the concentration range studied, with correlation coefficients between 0.9970 and 0.9999. To determine the quality of the method, a recovery study was performed on over spiked replicates of blank blood samples. The method validation studies were performed at 1–100 ng/mL for blood samples, depending on the pesticide type, which showed ranging from 64% to 109.7 of recovery. Control experiments with reagents had been carried out before the analysis. The analytical parameters of the quantified GC analytes included in Σ_4_OCPs and Σ_4_PCBs are provided in Supplementary Table S2. For clarity, only the analytical parameters of the compounds included in the summary exposure variables reported in Table 1 are presented in the Supplementary Materials. To improve transparency, the summary exposure variables were defined as the sums of the quantified analytes reported in the final dataset. Σ_4_OPPs was calculated as the sum of chlorpyrifos-ethyl, acetochlor, phenthoate, and cypermethrin. Σ_4_PCBs was calculated as the sum of PCB101, PCB153, PCB202, and PCB180. Σ_4_OCPs was calculated as the sum of o,p’-DDE, α-HCH, o,p’-DDT, and p,p’-DDT. β-HCH was not detected in either group and was therefore not included in Σ_4_OCPs. Detection frequencies (above LOQ) for the analytes included in the Σ summary variables are provided in Supplementary Table S4.

### 2.6. ELISA Analysis

The blood samples were taken from participants in the morning following 12 h of fasting. The blood samples were centrifuged for 10 min at 3000 rpm, after which the supernatant was quickly removed and kept frozen at −80 °C until the assays were performed. Serum PON1 (cat.no E2157Hu, Bioassay Technology Laboratory, Shanghai, China) level was measured with commercially available enzyme-linked immune sorbent assay (ELISA) kits (standard curve range 5–600 ng/L) according to the manufacturer’s instructions. Optical density values for samples and standard samples were detected on Thermo Scientific (Waltham, MA, USA) Multiscan Go Microplate Reader ELISA reader at 450 nm. The results are presented as ng/mL.

### 2.7. Statistical Analysis

The data was evaluated statistically by IBM SPSS Statistics Version v25.0 (IBM Corp, Armonk, NY, USA). The normality of the data was assessed by one-sample Kolmogorov–Smirnov test. The mean (± standard deviation) describes the continuous variables such as age and pesticide levels; e.g., Mann–Whitney U test or independent sample *t*-test was applied depending on the normal distribution to investigate the mean difference between the patient and control groups. ROC analyses were performed as exploratory analyses. AUC values with 95% confidence intervals, together with cut-off values, sensitivity, and specificity, are presented in Supplementary Table S3. Statistical significance was defined as a two-sided *p*-value < 0.05.

## 3. Results

The results of the demographic data and analyzes of the obesity and control group in the study are given in Table 1. In the obesity group, BMI, glucose, AST, ALT, insulin, cortisol, total cholesterol, LDL, triglyceride, and HOMA-IR values were significantly higher and HDL levels were significantly lower (*p* < 0.05) than in control group. As a result of ELISA analysis, PON1 value was found to be significantly lower in the obesity group (102.8 ± 12.49 ng/mL) compared to the control group (134.8 ± 14.29 ng/mL) (*p* < 0.001). According to the toxicology analysis results, in the obese group, the levels of Σ_4_OPPs as 1.29 ± 1.33 ng/mL, Σ_4_OCPs as 40.76 ± 55.89 ng/mL, and Σ_4_PCBs as 121.53 ± 90.86 ng/mL were determined in blood samples. Based on these results, Σ_4_OPPs and Σ_4_PCBs values were found to be statistically significantly higher compared to the control group (*p* < 0.001) (Table 1). A negative correlation was observed between Σ_4_OPPs and Σ_4_PCBs with PON1 according to Spearman’s rho test (r: −0.392, *p* < 0.0001; r: −0.585, *p* < 0.0001, respectively) (Table 2).

**Figure 1:**
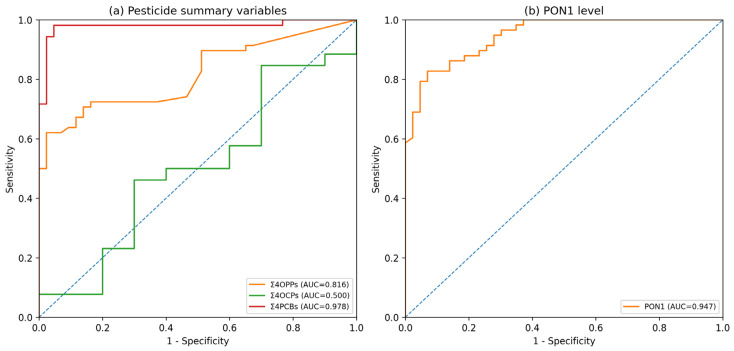
Receiver operating characteristic (ROC) curves for the discrimination of obese and control children. (**a**) ROC curves for the pesticide summary variables Σ_4_OPPs, Σ_4_OCPs, and Σ_4_PCBs. (**b**) ROC curve for PON1 level. AUC values are shown in the legends.

ROC analyses were performed as exploratory analyses. Among the exposure-related variables, Σ_4_PCBs showed the best discriminatory performance between obese and control children (AUC: 0.978, 95% CI: 0.944–1.000), followed by PON1 level (AUC: 0.947, 95% CI: 0.901–0.980). Σ_4_OPPs also showed good discriminatory ability (AUC: 0.816, 95% CI: 0.732–0.888), whereas Σ_4_OCPs showed no meaningful discrimination in this dataset (AUC: 0.500, 95% CI: 0.285–0.727) (Figure 1; Supplementary Table S3). Among the routine clinical variables, BMI showed perfect discrimination (AUC: 1.000, 95% CI: 1.000–1.000), while total cholesterol, triglycerides, and HOMA-IR showed moderate-to-good discriminatory performance (AUCs: 0.868, 0.817, and 0.855, respectively). HDL and LDL showed lower discriminatory performance (AUCs: 0.704 and 0.659, respectively) (Supplementary Table S3).

## 4. Discussion

Upon evaluating all the data obtained from our analyses, we found that the PON1 level was significantly lower in the obesity group (102.8 ± 12.49 ng/mL) (*p* < 0.001), as determined by ELISA analysis. LC/MS/MS and GC analyses showed elevated levels of Σ_4_OPPs and Σ_4_PCBs in obese children (*p* < 0.001), whereas no significant difference was detected between the two groups regarding Σ_4_OCPs (*p* > 0.05).

PON1 is an enzyme associated with HDL in serum that plays a key role in the detoxification of certain OPPs and contributes to antioxidant defense by hydrolyzing oxidized lipids. The levels and activity of PON1 are influenced by genetic and environmental factors, and its activity can vary across individuals and ethnic groups [22]. It should be noted that, in the present study, PON1 concentration was measured by ELISA, whereas enzymatic activity was not directly assessed. Therefore, PON1 levels should not be interpreted as a direct surrogate of functional detoxification capacity. In addition, PON1 polymorphisms such as Q192R and L55M may affect enzymatic activity independently of circulating protein levels. One of our main findings is that lower PON1 levels in obese children were observed together with increased insulin and HOMA-IR values, indicating an unfavorable metabolic profile. Furthermore, lower PON1 levels were accompanied by higher cholesterol and triglyceride levels and lower HDL levels, which may be associated with an increased cardiometabolic risk profile. Similar findings were reported by Ferré et al. [23]. Additionally, Huen et al. reported an association between PON1 and obesity-related risk in children [24]. Another noteworthy finding was the significantly elevated levels of Σ_4_OPPs and Σ_4_PCBs in obese children, together with the observed negative correlations of these exposure groups with PON1. In particular, the marked increase in phenthoate among OPPs and in PCB101 and PCB180 among PCBs were notable. Pesticides may increase the number and size of adipocytes during critical developmental periods and/or throughout life, thereby enhancing lipid storage and altering adipose tissue function. Due to their effects on food intake and metabolism, which can lead to obesity and metabolic syndrome, they are referred to as “obesogenic” [25]. Recent evidence further supports the biological plausibility of this relationship. A recent review emphasized that endocrine disruptors, including persistent organic pollutants and organophosphorus pesticides, may promote adipogenesis, disrupt glucose and lipid metabolism, alter energy homeostasis, and induce a proinflammatory milieu, thereby increasing susceptibility to obesity and metabolic disorders; however, the authors also highlighted that prenatal and postnatal human data are still insufficient in drawing definitive conclusions [26]. In parallel, a recent study in children environmentally exposed to pesticides reported significantly higher dialkylphosphate metabolites, malondialdehyde, 8-hydroxy-2′-deoxyguanosine, and IL-8 levels, supporting oxidative stress, DNA damage, and inflammation as relevant pathways of pesticide-related health effects in childhood [27]. Similarly, another recent study showed that children indirectly exposed to organophosphate pesticides had lower GH and IGF-1 levels and reduced cholinesterase values together with growth-related alterations, suggesting that chronic low-level exposure may affect endocrine and developmental processes in pediatric populations [28]. In addition, the higher blood levels of lipophilic compounds such as OCPs and PCBs observed in obese children may partly reflect increased adipose tissue mass and passive bioaccumulation rather than a direct causal or mechanistically specific role in obesity. Therefore, these findings should be interpreted cautiously, and the cross-sectional design of the present study does not allow discrimination between storage-related accumulation and biologically active exposure effects. Taken together, these recent findings do not establish a causal role of pesticide exposure in childhood obesity; rather, they strengthen the biological plausibility that pesticide-related oxidative, inflammatory, and endocrine perturbations may accompany obesity-related metabolic changes [26,27,28].

In the literature, there are few studies that examine pesticide exposure (particularly OPPs and PCBs) and PON1 levels simultaneously in obese children. It is well known that PON1 plays a key role in the detoxification of certain OPPs. Polymorphisms in the PON1 gene at positions Q192R and L55M influence paraoxonase activity [29]. In a study involving 373 young Mexican-American children aged two and five years living in an agricultural community, the likelihood of obesity was found to be 9.3 and 2.5 times higher, respectively, in children with the PON1 192QQ genotype compared to those with the PON1 192RR genotype. These findings suggest that an increase in the number of PON1 192Q alleles may be associated with obesity risk in children, supporting the relevance of PON1 in obesity-related metabolic research [24]. In another study involving 325 children and their mothers, slightly lower PON1 activity was observed in 9-year-old children compared to their mothers. However, no significant association was found between PON1 activity and obesity in either the children or their mothers [30]. An important study utilizing microarray-based differential gene expression analysis examined a set of 162 genes between high- and low-PCB exposure groups. The results showed elevated PON1 expression in the high PCB exposure group. PON1 was identified as a critical enzyme involved in metabolizing oxidized lipids, thereby protecting against vascular diseases and enhancing the body’s antioxidant defense. Furthermore, molecules such as PON1, BCL2, and ITGB1 were identified as potential signature biomarkers of PCB exposure [31].

An additional finding of this study is the exploratory ROC analysis (Figure 1; Supplementary Table S3). Among the exposure-related variables, Σ_4_PCBs showed the strongest discriminatory performance, followed by PON1 level and Σ_4_OPPs, whereas Σ_4_OCPs showed no clear discriminatory value in this dataset. However, these ROC findings should be interpreted cautiously. Because the present study was cross-sectional and based on a relatively small sample, the reported AUCs and cut-off values should be considered exploratory rather than clinically validated diagnostic thresholds. In addition, ROC analyses for BMI and routine metabolic markers are of limited independent clinical relevance in a predefined obese versus control comparison, and the apparently perfect discrimination observed for BMI mainly reflects the case-definition structure of the study. Therefore, the ROC findings for pesticide summary variables and PON1 should be viewed as preliminary and hypothesis-generating. This study has several limitations that should be considered when interpreting the findings. First, the relatively small sample size may limit the generalizability of the results to broader populations. Second, the study did not account for potential confounding factors such as dietary habits, physical activity levels, socioeconomic status, residential proximity to agricultural areas, parental occupation, or other environmental exposures that could influence both pesticide levels and obesity. These unmeasured factors may have biased the observed associations, and residual confounding cannot be excluded. Finally, the assessment of pesticide levels was limited to blood samples and may not fully capture long-term or cumulative exposure, which can be more accurately assessed through adipose tissue or urine samples. These limitations highlight the need for more comprehensive, large-scale, and longitudinal studies to build on these findings.

## 5. Conclusions

Our findings indicate the presence of OPPs and PCBs in the blood of obese children, underscoring the need for a thorough assessment of exposure risk factors. Lower PON1 levels were observed in obese children together with higher pesticide group levels and altered lipid parameters; however, the cross-sectional design does not allow conclusions regarding directionality or causation. Larger population-based and longitudinal studies are required to clarify the relationships among OPP and PCB levels, PON1, and obesity-related metabolic alterations, as well as to determine whether these associations are reproducible in broader pediatric populations.

## Figures and Tables

**Table 1:** Statistical comparison of biochemical and clinical parameters, as well as blood pesticide levels (ng/mL) in study groups.

	Study Groups		*p*-Value
	Patient	Control	
	Mean ± SD (Median)	Mean ± SD (Median)	
Age (years)	10.05 ± 3.54 (9.00)	9.58 ± 2.72 (9.00)	0.469
BMI	29.00 ± 1.49 (28.90)	21.34 ± 1.51 (21.45)	<0.001
Glucose	88.33 ± 7.42 (88.00)	79.28 ± 5.78 (79.00)	<0.001
AST (mg/dL)	27.76 ± 16.69 (25.00)	21.00 ± 3.39 (20.00)	0.004
ALT (mg/dL)	27.17 ± 30.29 (21.50)	16.58 ± 5.31 (15.00)	<0.026
GGT (mg/dL)	21.87 ± 12.08 (21.00)	18.12 ± 6.53 (19.00)	0.048
Insulin (mg/dL)	12.84 ± 6.35 (11.40)	6.90 ± 1.79 (7.00)	<0.001
Cortisol (mg/dL)	9.72 ± 3.03 (9.35	7.57 ± 2.91 (7.10)	0.001
Total cholesterol (mg/dL)	159.6 ± 22.0 (159.0)	125.8 ± 21.31 (131.0)	<0.001
HDL (mg/dL)	42.11 ± 7.40 (42.00)	46.62 ± 5.65 (48.00)	0.001
LDL (mg/dL)	90.32 ± 17.24 (91.80)	83.46 ± 7.87 (80.50)	0.009
Triglyceride (mg/dL)	122.3 ± 60.75 (103.5)	73.29 ± 19.52 (72.00)	<0.001
TSH (mg/dL)	2.13 ± 0.91 (2.10)	2.32 ± 0.76 (2.21)	0.276
T4 (mg/dL)	1.04 ± 0.17 (1.01)	1.11 ± 0.15 (1.09)	0.002
HOMAIR	2.83 ± 1.48 (2.35)	1.36 ± 0.41 (1.35)	<0.001
PON1 (ng/mL)	102.8 ± 12.49 (101.0)	134.8 ± 14.29 (135.8)	<0.001
Ʃ_4_OPPs (ng/mL)	1.29 ± 1.33 (0.87)	0.22 ± 0.20 (0.27)	<0.001
Chlorpyrifos-Ethyl (ng/mL)	0.37 ± 0.16 (0.28)	0.28 ± 0.08 (0.29)	0.891
Acetochlor (ng/mL)	0.29 ± 0.24 (0.20)	0.21 ± 0.12 (0.17)	0.523
Phenthoate (ng/mL)	1.21 ± 0.47 (1.06)	0.20 ± 0.05 (0.17)	0.001
Cypermethrin (ng/mL)	1.15 ± 1.32 (0.43)	0.50 ± 0.53 (0.50)	0.503
Ʃ_4_OCPs (ng/mL)	40.76 ± 55.89 (21.24)	30.49 ± 24.10 (20.87)	0.447
opDDE (ng/mL)	27.67 ± 15.07 (26.32)	15.17 ± 11.43 (9.15)	0.225
alfaHCH (ng/mL)	13.27 ± 4.60 (12.58)	14.62 ± 4.29 (14.62)	0.824
BetaHCH (ng/mL)	Nd	Nd	*
opDDT (ng/mL)	18.18 ± 7.91 (17.55)	23.94 ± 0.00 (23.94)	0.543
ppDDT (ng/mL)	155.2 ± 116.4 (216.4)	32.23 ± 24.96 (21.03)	0.180
Ʃ_4_PCBs (ng/mL)	121.53 ± 90.86 (122.4)	7.88 ± 4.25 (8.12)	<0.001
PCB101 (ng/mL)	17.47 ± 2.58 (17.13)	2.80 ± 0.97 (2.77)	<0.001
PCB153 (ng/mL)	60.86 ± 48.54 (53.62)	19.67 ± 0.00 (19.67)	0.439
PCB202 (ng/mL)	15.49 ± 16.76 (6.98)	7.77 ± 0.00 (7.77)	0.728
PCB180 (ng/mL)	136.5 ± 55.14 (120.4)	5.87 ± 1.70 (5.80)	<0.001

Nd: not detected, *p* < 0.05: independent sample *t*-test, * statistical analysis could not be performed between groups with undetectable values. Σ_4_OPPs was calculated as the sum of chlorpyrifos-ethyl, acetochlor, phenthoate, and cypermethrin; Σ_4_OCPs as the sum of o,p’-DDE, α-HCH, o,p’-DDT, and p,p’-DDT; and Σ_4_PCBs as the sum of PCB101, PCB153, PCB202, and PCB180. β-HCH was not detected in either group and was therefore not included in Σ_4_OCPs.

**Table 2:** Correlation between PON1, Ʃ_4_OPs, Ʃ_4_OCs, and Ʃ_4_PCBs values in the obese group.

			PON1	Ʃ_4_OPPs	Ʃ_4_OCPs	Ʃ_4_PCBs
**Spearman’s rho**	**PON1**	**r**	1.000	−0.392 **	−0.035	−0.585 **
		**p**	-	0.000	0.839	0.000
	**Ʃ_4_OPPs**	**r**	−0.392 **	1.000	0.097	0.430 **
		**p**	0.000	-	0.575	0.000
	**Ʃ_4_OCPs**	**r**	−0.035	0.097	1.000	0.009
		**p**	0.839	0.575	-	0.962
	**Ʃ_4_PCBs**	**r**	−0.585 **	0.430 **	0.009	1.000
		**p**	0.000	0.000	0.962	-

**: Correlation is significant at the 0.01 level.

## Data Availability

The original contributions presented in this study are included in the article. Further inquiries can be directed to the corresponding authors.

## Supplementary Materials

The following supporting information can be downloaded at https://www.mdpi.com/article/10.3390/biom16030439/s1. Table S1. Analysis parameters for LC-MS/MS device of organophosphorus pesticides (OPPs). Table S2. Analysis parameters for GC instrument of organochlorine pesticides (OCPs) and polychlorine biphenyls (PCBs). Table S3. Exploratory ROC performance of anthropometric, metabolic, exposure-related, and PON1 variables for discrimination between obese and control children (AUC values with 95% confidence intervals, cut-off values, sensitivity, and specificity). Table S4. Detection frequency (above LOQ) of analytes included in the Σ summary exposure variables.
