# Supplementary material for: Determination of Pesticide Residue Levels and Serum Paraoxonase 1 Protein Levels in Obese Children: A Case–Control Study
Source: Biomolecules. 2026 Mar 14;16(3):439. doi: 10.3390/biom16030439 (PMC13024204; doi:10.3390/biom16030439)
Supplement: Supplementary file 1 [file biomolecules-16-00439-s001.zip › biomolecules-4162661-supplementary.pdf]

Supplementary Materials

Table S1. Analytical parameters of LC-MS/MS analytes included in Σ4OPPs.

OPPs	Pesticide Class	RT (min.)	Precursor Ion (m/z)	Production -1 (m/z)	Production -2 (m/z)
Fenoxaprop-P-Ethyl	Herbicide	13.491	361.800	288.100	91.250
Triclorfon	Insecticide	7.186	256.850	109.000	220.700
Cymoxanil	Fungicide	8.442	199.100	128.150	111.100
Chlormequat Chloride	Herbicide	0.909	122.000	58.200	63.100
Clothianidine	Insecticide (Neonicotinoid)	7.338	249.700	132.100	169.100
Dimethoate	Insecticide	7.849	229.600	125.150	199.050
Acetamiprid	Neonicotinoid Insecticide	7.992	222.600	56.200	126.100
Chloridazon	Herbicide	7.990	221.600	104.100	92.200
Dichlorfos	Insecticide(Organophosphate)	10.300	220.900	109.150	127.150
Carbaryl	Insecticide(Carbamate)	10.776	202.050	145.200	127.150
Ethiofencarb	Insecticide(Carbamate)	10.957	225.800	107.150	164.200
Carboxin	Anilide Fungicide	10.754	235.600	143.100	87.100
Flutriafol	Fungicide	10.643	302.100	123.200	109.200
Atrazine	Herbicide(Triazine)	10.647	215.650	174.150	43.150
Diuron	Herbicide(Phenylureas)	10.872	232.800	72.200	160.100
Diphenamid	Herbicide	10.936	239.700	134.200	167.200
Diethofencarb	Fungiside(Carbamate)	11.896	267.800	226.200	180.200
Azinphos-Methyl	Insecticide(Organophosphate)	11.463	317.700	160.150	132.150
Azoxystrobin	Fungicide	11.696	403.800	372.250	329.100
Fenamidone	Foliar Fungicide	11.994	311.800	92.200	236.200
Boscalid	Anilide Fungicide	11.568	343.100	307.200	140.000
Dimethomorph	Fungicide	11.679	387.700	301.150	165.200
Dimethenamid	Herbicide	11.526	275.600	244.200	168.200
Cyproconazole	Fungicide(Triazole)	12.035	292.100	70.200	125.150
Bromuconazole	Fungicide(Triazole)	12.399	378.000	159.000	161.000
Fenbuconazole	Fungicide(Triazole)	12.633	337.150	70.250	125.100
Fenarimol	Fungicide(Pyrimidine)	12.499	330.800	268.100	139.100
Dichlofluanid	Fungicide(Sulfamide)	12.495	350.000	224.050	123.200
Epoxiconazole	Fungicide(Azole)	12.598	329.800	101.200	121.200
Alachlor	Herbicide(Chloroacetanilide)	12.737	269.700	238.250	162.250
Fenamiphos	Organophosphate Insecticide	12.338	303.600	217.100	202.100
Acetochlor	Herbicide	12.664	269.900	224.200	148.200
Buprimate	Pyrimidine Fungicide	12.783	316.700	166.200	108.100
Eptc	Herbicide	12.982	190.100	86.000	128.200
Fenthion	Insecticide(Thiophosphate)	13.108	279.000	247.100	169.000
Diniconazole	Fungicide	13.349	326.100	70.200	159.100
Diazinon	Insecticide(Pyrimidine)	13.093	304.600	169.100	97.100
Difenacozole	Fungicide(Triazole)	13.326	405.800	251.100	111.100
Cyprodinil	Fungicide	13.262	225.700	93.150	108.200
Cycloate	Herbicide	13.554	216.150	154.200	134.200
Diclofop Methyl	Herbicide	13.792	358.100	281.100	120.200
Dioxathion	Organophosphate Insecticide	13.608	473.800	97.100	153.100
Buprofezin	Insecticide	13.774	305.700	57.150	201.200
Diafenthiuron	Insecticide	14.123	384.900	329.300	278.250
Fenpropathrin	Insecticide(Pyrethroid)	14.063	350.150	125.200	57.200
Chlorfluazuron	Insecticide(Benzoylurea)	14.197	539.800	382.950	158.150
Cypermethrin	Insecticide(Pyrethroid)	14.182	433.300	191.050	193.150
Deltamethrin	Insecticide(Pyrethroid)	14.301	522.800	281.000	506.200

Abamectin	Insecticide	14.462	890.600	305.350	145.150
Fenpyroximate	Acaricide(Pyrazole)	14.286	421.700	366.300	135.100
Carbosulfan	Insecticide(Carbamate)	14.783	380.700	118.250	160.250
Fenazaquin	Acaricide(Quinazoline)	14.834	306.900	57.200	161.200
Bifenthrin	Insecticide(Pyrethroid)	15.109	440.200	181.200	166.100
Etofenprox	Insecticide(Pyrethroid)	15.206	393.800	177.200	107.150
Bensulfuron-Methyl	Herbicide	11.383	410.800	149.200	182.200
Clodinafob-Propargyl	Herbicide(Pyridine)	12.829	349.700	266.000	238.100
Ethofumesate	Herbicide	11.951	287.100	121.200	258.800
Ethoprophos	Organothiophosphate Insecticide	12.615	242.800	131.000	172.900
Chlorpyrifos-Ethyl	Organothiophosphate Insecticide	14.091	349.700	197.900	97.000
Chlorpyrifos-Methyl	Organothiophosphate Insecticide	13.532	321.900	125.000	289.900
Chlorsulfuron	Herbicide	7.757	357.800	141.200	167.200
Dazomet	Fumigant	4.921	163.000	89.900	119.900
Nicosulfuron	Herbicide(Pyridine)	6.753	411.100	182.200	106.200
Propamocarb Hydrochlo-ride	Fungicide	5.331	188.800	102.200	144.200
Oxamyl	Carbamate Pesticide	5.179	236.850	72.250	90.250
Methomyl	Insecticide(Carbamate)	5.666	162.800	88.200	106.200
Monocrotophos	Organophosphate Insecticide	6.361	224.100	127.100	98.200
Imidachloropid	Insecticide(Neonicotinoid)	7.170	256.100	209.150	175.150
Imazethapyr	Herbicide(Pyridine)	6.329	289.800	230.200	177.200
Foramsulfuron	Herbicide(Benzamide)	8.939	453.000	182.200	139.100
Oxadixyl	Anilide Fungicide	9.662	279.150	219.250	132.250
Monolinuron	Herbicide	10.204	215.000	126.100	148.200
Pirimicarb	Insecticide(Carbamate)	10.382	238.700	72.150	182.300
Metalaxyl M	Anilide Fungicide	11.378	279.750	220.250	192.250
Isoxaflutole	Herbicide(Isoxazole)	11.418	377.300	251.100	219.850
Methidathion	Organothiophosphate Insecticide	11.686	303.000	144.900	85.200
Phosmet	Organothiophosphate Insecticide	11.204	318.050	160.150	133.150
Myclobutanil	Fungicide(Triazole)	11.817	288.900	70.200	288.900
Malathion	Organothiophosphate Insecticide	12.240	330.800	127.200	99.100
Propyzamide	Herbicide(Benzamide)	12.325	256.050	190.050	173.050
Pyridaphenthion	Organothiophosphate Insecticide	12.321	340.800	189.150	92.200
Flusilazole	Organosilicon Fungicide	12.711	315.800	247.200	165.150
Metolachlor	Herbicide	12.733	283.800	252.200	176.250
Prometryn	Herbicide	12.642	241.800	158.100	200.200
Penconazole	Fungicide(Triazole)	12.958	284.250	70.250	159.050
Imazalil	Fungicide	12.953	297.100	159.000	255.100
Phenthoate	Organothiophosphate Insecticide	12.869	321.100	135.150	163.250
Kresoxim M	Fungicide	12.927	313.900	222.250	206.200
Pyraflufen-Ethyl	Herbicide(Pyrazole)	12.924	413.100	339.100	253.100
Metconazole	Fungicide(Triazole)	13.140	320.200	70.200	125.150
Hexaconazole	Fungicide(Triazole)	13.149	314.100	70.200	159.100
Propiconozole	Fungicide(Triazole)	12.789	342.100	159.000	69.200
Mefenpyr-Diethyl	Herbicide	13.104	389.900	327.150	160.000
Prochloraz	Fungicide(Imidazole)	13.204	375.800	308.100	266.000
Phosalone	Organothiophosphate Insecticide	13.195	368.050	182.150	111.100
Pyraclostrobin	Fungicide(Carbamate)	13.120	387.800	163.100	104.200
Pirimiphos-Methyl	Organothiophosphate Insecticide	13.092	305.800	108.100	164.200
Pyrazophos	Fungicide	13.286	373.800	222.200	194.200

Haloxyfop-2-Ethoxy Ethyl	Herbicide(Pyridine)	13.558	433.900	316.150	91.200
Fluazifop- P-Butyl	Herbicide	13.565	383.900	282.200	328.200
Profenofos	Organophosphate Insecticide	13.529	372.950	302.900	345.100
Propaquizafop	Herbicide	13.657	444.200	371.000	100.200
Hexythiazox	Acaricide	13.992	353.200	228.100	168.100
Propargite	Acaricide	14.064	368.250	175.200	231.300
Pyriproxyfen	Insecticide(Pyridine)	13.996	321.700	96.200	185.150
Pendimethalin	Herbicide(Aniline)	14.179	282.200	194.100	212.200
Pyridaben	Organochlorine Insecticide	14.448	365.000	147.300	309.200
Prothiophos	Organothiophosphate Insecticide	12.936	344.500	241.100	133.200
Pyridate	Herbicide(Pyridazine)	5.036	379.200	207.100	351.250
Halfenprox	Insecticide(Pyrethroid)	15.459	494.200	183.200	155.100
Fosthiazate	Organothiophosphate Insecticide	10.999	284.100	104.200	227.700
Furathiocarb	Insecticide(Carbamate)	13.652	382.900	195.200	252.100
Haloxyfop-P-Methyl	Insecticide(Pyridine)	13.337	376.100	315.900	91.200
Imazapyr	Herbicide(Pyridine)	3.710	261.900	217.200	78.200
Iodosulfuron-Methyl	Herbicide	9.480	508.000	167.200	141.200
Imazapic	Herbicide	8.270	275.900	231.100	163.200
Mesosulfuron-Methyl	Herbicide	9.826	504.200	182.200	139.200
Mesotrione	Herbicide	4.567	357.100	104.100	104.100
Metamitron	Herbicide(Triazine)	7.753	203.100	104.200	174.000
Metribuzin	Herbicide(Triazine)	9.480	215.100	187.100	84.300
Quizalofop-P-Ethyl	Herbicide	13.579	373.500	299.500	299.100
Spirodiclofen	Organochlorine Acaricide	4.829	411.200	71.200	313.000
Spiroxamine	Fungicide	12.908	298.000	144.300	100.300
Tau Fluvalinate	Insecticide(Pyrethroid)	14.358	520.000	181.200	208.000
Tebuconazole	Fungicide(Triazole)	12.955	308.200	70.200	125.200
Tebufenozide	Insecticide	12.725	353.300	133.200	296.900
Terbutryn	Herbicide(Triazine)	12.738	241.700	186.200	68.200
Tetraconazole	Fungicide(Triazole)	12.510	372.100	159.100	70.200
Thiacloprid	Insecticide(Neonicotinoid)	8.787	252.750	126.100	99.100
Thiamethoxam	Insecticide(Neonicotinoid)	5.988	291.800	210.900	181.200
Thifensulfuron-Methyl	Herbicide(Triazine)	7.028	388.100	167.200	69.100
Thiometon	Organothiophosphate Insecticide	10.467	247.050	89.200	88.800
Tolylfluanid	Fungicide(Sulfamide)	12.897	363.800	237.900	137.200
Triadimefon	Fungicide(Triazole)	12.286	294.100	197.000	70.200
Triasulfuron	Herbicide	7.943	402.100	167.200	141.100
Florasulam	Herbicide	6.961	360.050	129.100	192.000
Mesotrione	Herbicide	4.547	357.100	227.900	104.100
Thiophanate-Methyl-Pos	Fungicide(Carbamate)	9.453	343.050	151.100	118.200
Metsulfuron-Methyl	Herbicide(Sulfonylurea)	6.523	382.100	167.200	198.900
Metribuzin	Herbicide(Triazine)	9.474	214.800	187.000	84.100
Fenoxycarb	Insecticide(Carbamate)	12.801	302.150	88.200	116.100
Triflumizole	Fungicide(Imidazole)	13.507	346.500	278.200	73.000
Trifloxystrobin	Fungicide	13.341	409.150	186.200	145.100
Fipronil	Insecticide(Pyrazole)	12.654	434.900	329.900	249.9

RT: retention times (minute).

Table S2. Analytical parameters of GC analytes included in Σ4OCPs and Σ4PCBs.

	RT (min.)	Calibration ranges (ng/mL)
	**OCPs**	
alfa-HCH	14.520	5-100 ng/mL
HCB	14.858	1-100 ng/mL
β-HCH	15.745	10-100 ng/mL
δ - HCH	15.989	5-100 ng/mL
γ - HCH	17.164	5-100 ng/mL
o,p′-DDE	24.419	10-100 ng/mL
p,p′-DDE	24.566	1-100 ng/mL
o,p′-DDD	25.766	5-100 ng/mL
p,p′-DDD	26.048	5-100 ng/mL
o,p′-DDT	27.235	5-100 ng/mL
p,p′-DDT	28.276	10-100 ng/mL
	**PCBs**	
PCB-118	26.896	5-100 ng/mL
PCB-153	27.167	25-100 ng/mL
PCB-28	18.801	10-100 ng/mL
PCB-52	20.536	5-100 ng/mL
PCB-101	24.668	5-50 ng/mL
PCB-138	28.398	5-100 ng/mL
PCB-202	29.732	1-100 ng/mL
PCB-180	30.221	5-100 ng/mL

RT: retention times (minute).

Table S3. Exploratory ROC performance of anthropometric, metabolic, exposure-related, and PON1 variables for discrimination between obese and control children. AUC values with 95% confidence intervals, cut-off values, sensitivity, and specificity are presented.

Variable	n	AUC	95% CI	Cutoff (ng/mL)	Direction for Obesity Classification	Sensitivity (%)	Specificity (%)
BMI	101	1	1.000–1.000	26	higher values	100	100
Total cholesterol	101	0.868	0.797–0.931	152	higher values	65.5	100
HDL	101	0.704	0.596–0.795	44.8	lower values	72.4	72.1
LDL	101	0.659	0.547–0.758	96.5	higher values	41.4	100
Triglycerides	101	0.817	0.727–0.893	88	higher values	77.6	79.1
HOMA-IR	101	0.855	0.770–0.930	1.815	higher values	79.3	83.7
Σ4OCPs	36	0.5	0.285–0.727	28.79	higher values	46.2	70
Σ4OPPs	101	0.816	0.732–0.888	0.56	higher values	62.1	97.7
Σ4PCBs	96	0.978	0.944–1.000	12.88	higher values	98.1	95.3
PON1	101	0.947	0.901–0.980	112.4	lower values	82.8	93

Table S4. Detection frequency (above LOQ) of analytes included in the Σ summary exposure variables.

Summary Variable	Analyte	Obese (n/N, %)	Control (n/N, %)	Overall (n/N, %)
OPPs (Σ4OPPs)	Chlorpyrifos-ethyl	38/58 (65.5%)	24/43 (55.8%)	62/101 (61.4%)
OPPs (Σ4OPPs)	Acetochlor	15/58 (25.9%)	4/43 (9.3%)	19/101 (18.8%)
OPPs (Σ4OPPs)	Phenthoate	19/58 (32.8%)	3/43 (7.0%)	22/101 (21.8%)
OPPs (Σ4OPPs)	Cypermethrin	33/58 (56.9%)	2/43 (4.7%)	35/101 (34.7%)
OCPs (Σ4OCPs)	o,p′-DDE	11/58 (19.0%)	3/43 (7.0%)	14/101 (13.9%)
OCPs (Σ4OCPs)	α-HCH	15/58 (25.9%)	2/43 (4.7%)	17/101 (16.8%)
OCPs (Σ4OCPs)	o,p′-DDT	5/58 (8.6%)	1/43 (2.3%)	6/101 (5.9%)
OCPs (Σ4OCPs)	p,p′-DDT	3/58 (5.2%)	5/43 (11.6%)	8/101 (7.9%)
PCBs (Σ4PCBs)	PCB-101	49/58 (84.5%)	38/43 (88.4%)	87/101 (86.1%)
PCBs (Σ4PCBs)	PCB-153	8/58 (13.8%)	1/43 (2.3%)	9/101 (8.9%)
PCBs (Σ4PCBs)	PCB-202	3/58 (5.2%)	1/43 (2.3%)	4/101 (4.0%)
PCBs (Σ4PCBs)	PCB-180	37/58 (63.8%)	35/43 (81.4%)	72/101 (71.3%)

* Detection frequency is reported as the number and percentage of samples with quantifiable concentrations (non-missing values) in the dataset for each group (Obese n=58; Control n=43; Overall n=101). β-HCH was not detected in either group and was therefore not included in Σ4OCPs.
